# Circadian factors CLOCK and BMAL1 promote nonhomologous end joining and antagonize cellular senescence

**DOI:** 10.1093/lifemedi/lnae006

**Published:** 2024-02-04

**Authors:** Yu Chen, Xiaoyu Xu, Zhixi Chen, Lingjiang Chen, Ying Jiang, Zhiyong Mao

**Affiliations:** Shanghai Key Laboratory of Maternal Fetal Medicine, Clinical and Translational Research Center of Shanghai First Maternity and Infant Hospital, Frontier Science Center for Stem Cell Research, School of Life Sciences and Technology, Tongji University, Shanghai 200092, China; Shanghai Key Laboratory of Signaling and Disease Research, School of Life Sciences and Technology, Tongji University, Shanghai 200092, China; Shanghai Key Laboratory of Maternal Fetal Medicine, Clinical and Translational Research Center of Shanghai First Maternity and Infant Hospital, Frontier Science Center for Stem Cell Research, School of Life Sciences and Technology, Tongji University, Shanghai 200092, China; Shanghai Key Laboratory of Signaling and Disease Research, School of Life Sciences and Technology, Tongji University, Shanghai 200092, China; Shanghai Key Laboratory of Maternal Fetal Medicine, Clinical and Translational Research Center of Shanghai First Maternity and Infant Hospital, Frontier Science Center for Stem Cell Research, School of Life Sciences and Technology, Tongji University, Shanghai 200092, China; Shanghai Key Laboratory of Signaling and Disease Research, School of Life Sciences and Technology, Tongji University, Shanghai 200092, China; Shanghai Key Laboratory of Maternal Fetal Medicine, Clinical and Translational Research Center of Shanghai First Maternity and Infant Hospital, Frontier Science Center for Stem Cell Research, School of Life Sciences and Technology, Tongji University, Shanghai 200092, China; Shanghai Key Laboratory of Signaling and Disease Research, School of Life Sciences and Technology, Tongji University, Shanghai 200092, China; Shanghai Key Laboratory of Maternal Fetal Medicine, Clinical and Translational Research Center of Shanghai First Maternity and Infant Hospital, Frontier Science Center for Stem Cell Research, School of Life Sciences and Technology, Tongji University, Shanghai 200092, China; Shanghai Key Laboratory of Signaling and Disease Research, School of Life Sciences and Technology, Tongji University, Shanghai 200092, China; Shanghai Key Laboratory of Maternal Fetal Medicine, Clinical and Translational Research Center of Shanghai First Maternity and Infant Hospital, Frontier Science Center for Stem Cell Research, School of Life Sciences and Technology, Tongji University, Shanghai 200092, China; Shanghai Key Laboratory of Signaling and Disease Research, School of Life Sciences and Technology, Tongji University, Shanghai 200092, China; Tsingtao Advanced Research Institute, Tongji University, Qingdao 266071, China

**Dear Editor,**

Circadian rhythm controls the daily oscillations of gene expression, cellular biological processes, and individual behavior. The cell-autonomous circadian feedback loop comprises a panel of proteins, among which two positive transcriptional regulators, namely, brain and muscle ARNT-like 1 (BMAL1, also known as ARNTL) and circadian locomotor output cycles kaput (CLOCK), are recognized as the core components of the circadian clock [1]. The breakdown of circadian rhythm is linked with tumorigenesis by a large number of studies [1]. Interestingly, circadian rhythm has also been found to impact the aging process [2–5]. Jet-lag in aged mice increases the mortality rate [2], and genetic deficiency in either *Bmal1* [3] or *Clock* [4] leads to the development of aging-associated phenotypes in mice. Moreover, a recent study reported that BMAL1 antagonizes cellular senescence through repressing LINE1 retrotransposon in primates [5].

DNA damage repair plays a vital role in regulating the onset of aging [6]. Double-strand break (DSB) is considered the most serious type of DNA damage. Accumulating evidence has demonstrated that DSB repair capacity declines with increasing age, and defects in DNA repair machinery accelerate the aging process [6]. Intriguingly, several recent studies have shown that DSB repair is regulated by circadian factors, either through transcription-dependent [7] or -independent [8] mechanisms in cancer cells, indicating that a crosstalk exists between circadian rhythm and DNA repair, which are both aging-related pathways. The interconnection between DNA repair, especially DSB repair, and circadian rhythm during the onset of aging is an emerging field to be investigated, and will ultimately offer novel opportunities for active health intervention.

Although previous studies found that circadian factors promote DSB repair through homologous recombination (HR) in cancer cells, knowledge is lacking about whether they are engaged in the regulation of nonhomologous end joining (NHEJ), the predominant sub-pathway of DSB repair in normal somatic cells. Using our dual fluorescence-based reporter system [9] (Fig. 1A), we found that overexpression of both CLOCK and BMAL1 significantly promoted the repair of DSBs through NHEJ, by around 2-fold, in human skin fibroblasts (Fig. 1B and 1C). The NHEJ promotion effect was also confirmed by using the NHEJ-I9A system (Fig. S1A and S1B). The analysis of NHEJ repair fidelity demonstrated that CLOCK and BMAL1 did not significantly influence the average insertion size of the repair product. However, CLOCK overexpression decreased the average deletion size, and BMAL1 overexpression also showed a trend towards reducing the deletion size, although the effect was not statistically significant (Fig. S1C). Additionally, in line with previous findings in cancer cells [8], overexpression of CLOCK and BMAL1 also significantly potentiated HR repair (Fig. S1D and S1E).

**Figure 1.**
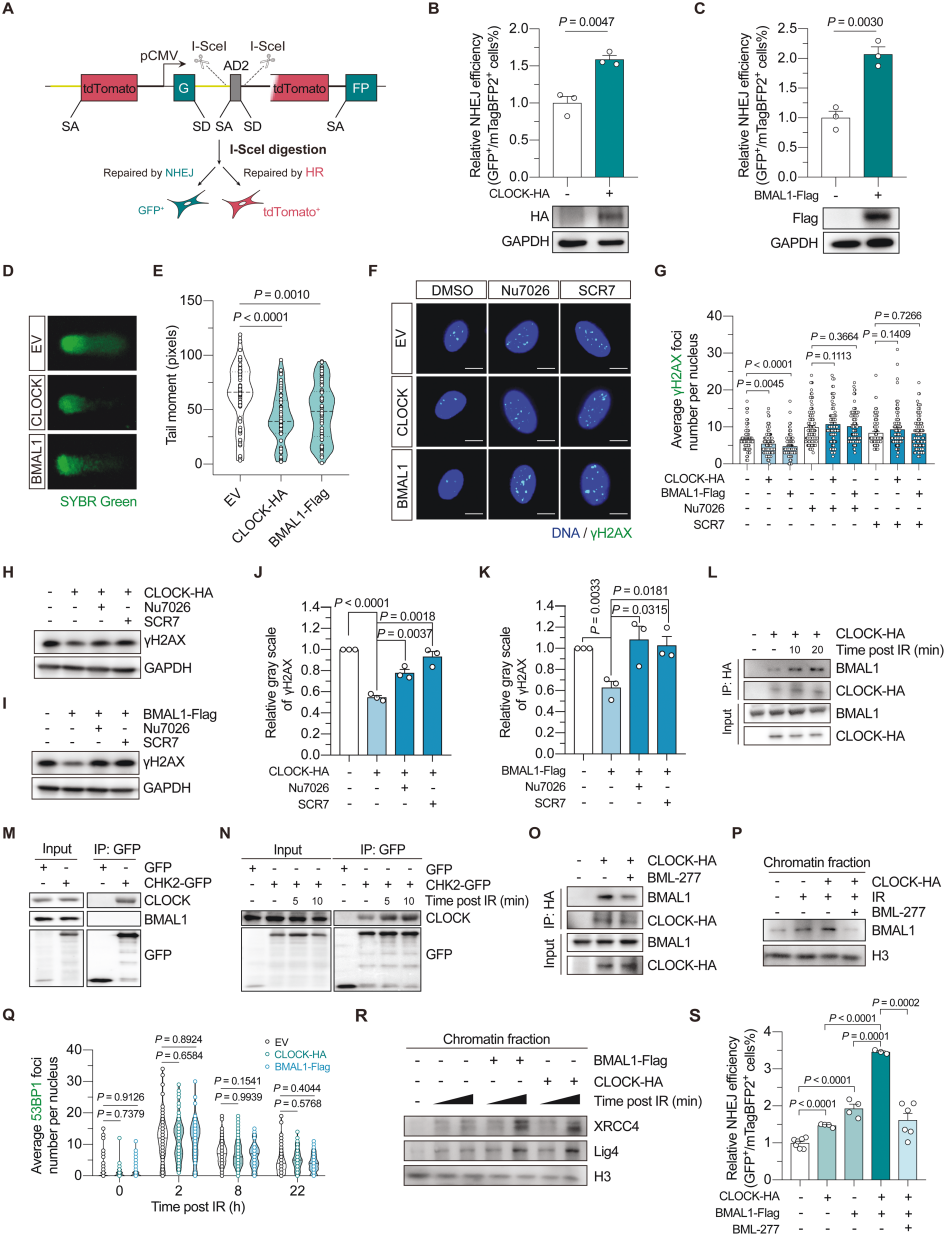
**CLOCK and BMAL1 collaborate to potentiate NHEJ repair by promoting XRCC4–Lig4 recruitment, which is dependent on CHK2 activity.**(A) Schematic diagram showing the dual-fluorescence reporter for HR-NHEJ efficiency analysis. (B) The effect of CLOCK overexpression on NHEJ efficiency. (C) The effect of BMAL1 overexpression on NHEJ efficiency. For (B) and (C), 5 μg of vectors encoding CLOCK or BMAL1 was transfected together with 5 μg of vectors encoding the I-SceI endonuclease and 15 ng of vectors encoding mTagBFP2 into HCA2-hTERT cells stably integrated with the dual-fluorescence reporter. (D, E) DNA damage in HCA2-hTERT cells introduced with vectors encoding CLOCK, BMAL1 or the empty vector (EV), assessed by comet assay. DNA was stained with SYBR green. Representative images are shown in (D), and the quantification results are shown in (E). (F, G) The average γH2AX foci number per nucleus in HCA2-hTERT cells 6 h post 2 Gy X-irradiation. Cells transfected with indicated vectors were pretreated with 7 μM Nu7026 or 50 μM SCR7 for 12 h before irradiation, and Nu7026 or SCR7 was re-supplemented into the culture medium post irradiation. Representative images are shown in (F) (scale bar: 10 μm), and data are quantified in (G). (H, I) Western blotting analysis of the γH2AX level in HEK293T cells at 4 h post 8 Gy X-irradiation. Cells were transfected with indicated vectors, and treated with either Nu7026 or SCR7 as described above. (J, K) Quantification of the Western blotting results of γH2AX. The gray scale of γH2AX was normalized to that of GAPDH. (L) The interaction between CLOCK and BMAL1 in HEK293T cells at the indicated time point post 8 Gy X-irradiation treatment. (M) Co-IP analysis of the interaction between CHK2 and the circadian factors in HEK293T cells. (N) Analysis of the CLOCK–CHK2 association in HEK293T cells at the indicated time point post 8 Gy X-irradiation treatment. (O) The effect of BML-277 treatment on CLOCK–BMAL1 interaction in HEK293T cells at 20 min post 8 Gy X-irradiation. (P) Western blotting analysis of the endogenous BMAL1 level in the chromatin fraction. Cells were transfected with vectors encoding CLOCK or the empty vector, then irradiated with or without 8 Gy X-irradiation. Chromatin fraction was extracted 2 h post X-irradiation. Cells were treated with BML-277 or the vehicle as indicated. (Q) Fluorescence immunostaining of 53BP1 foci in HCA2-hTERT cells at the indicated timepoint post 2 Gy X-irradiation. Cells were transfected with vectors encoding CLOCK, BMAL1 or the EV. (R) Western blotting analysis of the endogenous XRCC4 and Lig4 level in the chromatin fraction. Cells were transfected with indicated vectors before irradiation and subsequently harvested at 2 min or 10 min post-irradiation. (S) NHEJ efficiency analysis in cells transfected with 4 μg of vectors encoding CLOCK and/or 4 μg of vectors encoding BMAL1 (EV was co-transfected to ensure the total amount of vectors transfected was equal), with or without BML-277 treatment. Data are shown as mean ± SEM in column plots. Student’s *t* test for (B), (C), (J), (K), and (S), and Mann-Whitney *U* test for (E), (G), and (Q).

To further investigate whether CLOCK and BMAL1-mediated promotion of DSB repair stabilized the genome, we subsequently performed a comet assay in the human skin fibroblasts, HCA2-hTERT cells. The results showed that overexpressing CLOCK and BMAL1 significantly reduced the tail moment, indicating an improved genomic integrity (Fig. 1D and 1E). To verify that CLOCK and BMAL1-mediated NHEJ promotion is crucial for the maintenance of genomic integrity, immunofluorescence staining with an antibody against γH2AX was performed. The results showed that overexpression of CLOCK and BMAL1 significantly accelerated the clearance of DNA damage induced by ionizing radiation (IR) (Fig. 1F and 1G). However, blocking canonical NHEJ pathway by treating cells with either Nu7026, a DNA-PKcs inhibitor, or SCR7, a DNA Lig4 inhibitor, abolished the stimulatory effect of CLOCK and BMAL1 overexpression on the clearance of γH2AX foci (Fig. 1F and 1G). This suggested that CLOCK and BMAL1 stabilized the genome by promoting NHEJ repair, at least in the early stage after DNA damage was induced. Similar results were also obtained when western blotting assay was performed (Fig. 1H–K).

BMAL1 forms a heterodimer with CLOCK to regulate the expression of circadian rhythm-associated genes [1], and interestingly, a previous study has also shown that both BMAL1 and CLOCK colocalize with the γH2AX foci [8]. We implemented a laser micro-irradiation method to induce DNA damage, and found that both the BMAL1 and CLOCK were recruited to DNA damage sites (Fig. S1F and S1G). Co-IP experiment further demonstrated that the interaction between CLOCK and BMAL1 was enhanced post-IR (Fig. 1L). To gather further insights on whether factors involved in the upstream damage response mediate the CLOCK–BMAL1 association post the occurrence of DNA damage, co-IP analysis was performed, and the data showed that CLOCK, rather than BMAL1, interacted with the checkpoint kinase CHK2 (Figs. 1M, S2A and S2B). Of note, we found a stress-dependent enhancement of CLOCK–CHK2 association (Fig. 1N). Moreover, treating cells with BML-277, an inhibitor of the CHK2 kinase, diminished the enhancement of CLOCK–BMAL1 interaction post-IR (Fig. 1O). qPCR analysis was further conducted to eliminate the possibility that the function of CHK2 was exerted through influencing the canonical function of CLOCK and BMAL1 as transcription factors, since no obvious alterations were observed in the expression of their downstream genes (Fig. S3A–C). Then, the chromatin fractionation assay showed that the amount of chromatin-bound BMAL1 increased upon the occurrence of DNA damage (Fig. 1P). Moreover, overexpression of CLOCK could further promote BMAL1 loading onto chromatin while treating cells with BML-277 drastically disrupted the BMAL1 recruitment (Fig. 1P). Altogether, these data suggest that CHK2-mediated CLOCK–BMAL1 association is critical to BMAL1 recruitment after DNA is damaged.

To further decipher the molecular basis of CLOCK–BMAL1-mediated promotion of NHEJ repair, we first examined whether CLOCK and BMAL1 impacted 53BP1 recruitment kinetics. Nevertheless, the results showed that 53BP1 kinetics was not influenced by either CLOCK or BMAL1 overexpression (Figs. 1Q and S4). Subsequently, the chromatin fraction was extracted from HEK293T cells which were treated with IR, and subjected to Western blotting analysis. The data revealed that the recruitment of XRCC4 and DNA Lig4 was augmented when BMAL1 was overexpressed (Fig. 1R). In line with our finding above that CLOCK promoted the recruitment of BMAL1, we observed that overexpressing CLOCK exerted a similar promotional effect on the chromatin loading of both XRCC4 and DNA Lig4 (Fig. 1R). As a consequence, simultaneous overexpression of both CLOCK and BMAL1 further promoted NHEJ capacity, verifying that the two factors act cooperatively to regulate NHEJ repair, while BML-277 treatment significantly abolished the stimulatory effect (Figs. 1S and S5).

Unrepaired DNA damage is closely related to cellular senescence. To explore how the expression of BMAL1 or CLOCK changes in senescent cells, HCA2-hTERT cells were treated with IR to induce stress-induced premature senescence (SIPS). Western blotting analysis showed that the protein level of CLOCK, rather than BMAL1, dramatically decreased in senescent HCA2-hTERT cells (Fig. 2A). Moreover, this reduction in CLOCK protein level was attributed to a decline of *CLOCK* mRNA level (Fig. 2B). These data indicated that the CLOCK-BMAL1 axis-mediated regulation of NHEJ might be compromised in senescent cells. Thus, it intrigued us to study if overexpressing CLOCK or BMAL1 could delay the onset of SIPS (Fig. 2C). Senescence-associated β-galactosidase staining revealed that overexpressing either of the two factors had a beneficial effect in preventing the onset of SIPS (Fig. 2D and 2E). RT-qPCR data further confirmed that the mRNA level of *CDKN1A* was significantly reduced in CLOCK and BMAL1 overexpressed cells, indicating an amelioration of cellular senescence (Fig. 2F). Importantly, we found that overexpression of CLOCK and BMAL1 significantly attenuated senescence-associated secretory phenotype (SASP) by reducing the expression of *IL6*, *CCL2,* and *CXCL2* (Fig. 2G–I). CLOCK overexpression also contributed to a reduction in the expression of additional two signature SASP factors, *IL1b* and *IL8* (Fig. S6A–C), which in accordance with our finding that CLOCK is a possible rate-limiting factor protecting against the onset of SIPS in HCA2-hTERT cells.

**Figure 2.**
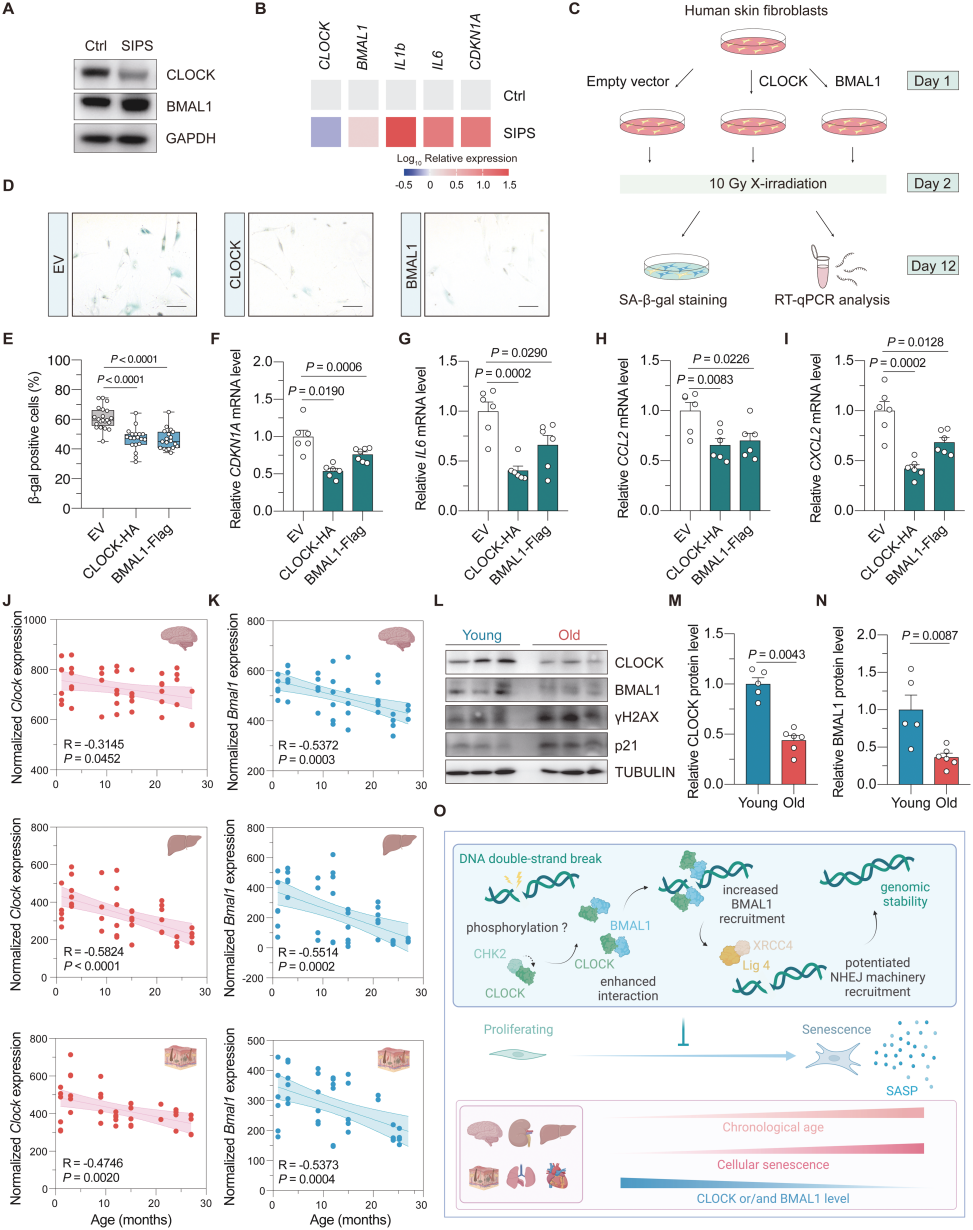
**Overexpression of CLOCK and BMAL1 counteracts stress-induced cellular senescence.**(A) Immunoblot of CLOCK and BMAL1 in stress-induced premature cellular senescent (SIPS) HCA2-hTERT cells. (B) Heat map showing the mRNA level of the indicated genes in proliferating and SIPS HCA2-hTERT cells. (C) Illustration of the experimental design. Vectors encoding CLOCK or BMAL1 were introduced into HCA2-hTERT cells, followed by 10 Gy X-irradiation treatment. On day 10 post irradiation, cells were subjected to senescence associated β-galactosidase (SA-β-gal) staining or RT-qPCR analysis. (D, E) SA-β-gal staining of cells transfected with CLOCK or BMAL1. Representative images are shown in (D) (scale bar: 100 μm), and data are quantified in (E). (F) RT-qPCR analysis of the mRNA level of *CDKN1A* upon CLOCK or BMAL1 overexpression. (G–I) The effect of CLOCK or BMAL1 overexpression on the mRNA levels of the indicated SASP genes. (J, K) Age-associated change of *Clock* and *Bmal1* mRNA levels in multiple mouse tissues. The data was extracted from Tabula Muris Senis database, and fitted using the linear regression model. (L–N) Western blotting analysis of the protein level of CLOCK and BMAL1 in the kidney of young (3-month-old) versus old (24-month-old) mice. Each dot represents for one mouse. (O) Working model. Upon the occurrence of DNA DSB, CHK2 interacts with CLOCK and promotes CLOCK–BMAL1 interaction, which depends on the kinase activity of CHK2. CLOCK subsequently facilitates the recruitment of BMAL1 to the DNA damage site, where they facilitate the recruitment of NHEJ machineries to promote NHEJ repair. However, the expression levels of CLOCK and/or BMAL1 decline during the onset of SIPS and chronological aging. Overexpression of CLOCK or BMAL1 antagonizes the onset of SIPS and attenuates the expression levels of SASP factors. Student’s *t* test for (F–I), and Mann–Whitney *U* test for (E), (M), and (N). Data are presented as mean ± SEM.

To investigate whether the BMAL1 and CLOCK-mediated regulation of NHEJ repair might be altered during the process of organ aging, we performed data mining on the RNA-seq resource available in Tabula Muris Senis database. *In silico* analysis demonstrated that the mRNA levels of both mouse *Bmal1* and *Clock* significantly declined with the increasing age in multiple organs, including the brain, liver, skin, lung, and kidney (Figs. 2J and 2K, S7A and S7B). Interestingly, the decrease in *Bmal1* expression level was observed in a broader spectrum of mouse tissues (Fig. S7B). To experimentally validate the RNA-seq data, kidney tissues were dissected from 3-month-old (young) and 24-month-old (physiologically aged) mice. Consistently, we found that both BMAL1 and CLOCK protein levels were significantly decreased in the kidney tissues of aged mice relative to those of young mice, accompanied by an increase in the γH2AX level (Figs. 2L–N and S7C), suggesting that dysregulations in circadian rhythm might be one of the underlying mechanisms of age-associated increase of genomic instability in the kidney of mice. An analogous pattern of age-related changes was also observed in the intestine (Fig. S7D and S7E).

Collectively, our study provided new insights into how the circadian rhythm and DNA repair interact during the aging process (Fig. 2O). Furthermore, it may also lay the foundation for future research on developing strategies that target circadian factors to promote healthy aging.

## Research limitations

Our study delineated that CHK2 played a crucial role in regulating CLOCK-BMAL1 heterodimer formation under genotoxic conditions. However, whether CHK2 directly phosphorylated CLOCK deserves further study. Moreover, the regulatory mechanisms underlying the downregulation of CLOCK and/or BMAL1 in a cell- or tissue-specific manner remain enigmatic. In addition, whether the alteration of CLOCK and BMAL1 expression contributes to age-associated NHEJ impairment also requires thorough investigation with the aids of *in vivo* DNA repair reporter models [10].

## Supplementary Material

lnae006_suppl_Supplementary_Figures_S1-S7

## References

[1] Masri S, Sassone-Corsi P. The emerging link between cancer, metabolism, and circadian rhythms. Nat Med 2018;24:1795–803. 30523327 10.1038/s41591-018-0271-8 PMC6535395

[2] Davidson AJ, Sellix MT, Daniel J, et al. Chronic jet-lag increases mortality in aged mice. Curr Biol 2006;16:R914–916. 17084685 10.1016/j.cub.2006.09.058 PMC1635966

[3] Kondratov RV, Kondratova AA, Gorbacheva VY, et al. Early aging and age-related pathologies in mice deficient in BMAL1, the core component of the circadian clock. Genes Dev 2006;20:1868–73. 16847346 10.1101/gad.1432206 PMC1522083

[4] Dubrovsky YV, Samsa WE, Kondratov RV. Deficiency of circadian protein CLOCK reduces lifespan and increases age-related cataract development in mice. Aging 2010;2:936–44. 21149897 10.18632/aging.100241 PMC3034182

[5] Liang C, Ke Q, Liu Z, et al. BMAL1 moonlighting as a gatekeeper for LINE1 repression and cellular senescence in primates. Nucleic Acids Res 2022;50:3323–47. 35286396 10.1093/nar/gkac146 PMC8989534

[6] Chen Y, Geng A, Zhang W, et al. Fight to the bitter end: DNA repair and aging. Ageing Res Rev 2020;64:101154. 32977059 10.1016/j.arr.2020.101154

[7] Shafi AA, McNair CM, McCann JJ, et al. The circadian cryptochrome, CRY1, is a pro-tumorigenic factor that rhythmically modulates DNA repair. Nat Commun 2021;12:401. 33452241 10.1038/s41467-020-20513-5 PMC7810852

[8] Zhang CF, Chen L, Sun L, et al. BMAL1 collaborates with CLOCK to directly promote DNA double-strand break repair and tumor chemoresistance. Oncogene 2023;42:967–79. 36725890 10.1038/s41388-023-02603-y PMC10038804

[9] Chen Y, Zhang H, Xu Z, et al. A PARP1-BRG1-SIRT1 axis promotes HR repair by reducing nucleosome density at DNA damage sites. Nucleic Acids Res 2019;47:8563–80. 31291457 10.1093/nar/gkz592 PMC7145522

[10] Chen Y, Cui Z, Chen Z, et al. IDDoR: A novel reporter mouse system for simultaneous and quantitative in vivo analysis of both DNA double-strand break repair pathways. Protein Cell 2023;14:369–75. 37155317 10.1093/procel/pwac001 PMC10166171

