# Supplementary material for: Circadian factors CLOCK and BMAL1 promote nonhomologous end joining and antagonize cellular senescence
Source: Life Med. 2024 Feb 4;3(2):lnae006. doi: 10.1093/lifemedi/lnae006 (PMC11749561; doi:10.1093/lifemedi/lnae006)
Supplement: lnae006_suppl_Supplementary_Figures_S1-S7 [file lnae006_suppl_Supplementary_Figures_S1-S7.zip › Supplementary_Figure and infomation_ready for typesetting_clean/Supplementary_infomation_ready for typesetting_clean.pdf]

Supplementary Information for "*Circadian factors CLOCK and BMAL1 promote nonhomologous end joining and antagonize cellular senescence*"

METHODS DETAILS

Cell culture and transfection

Human fibroblasts (HCA2-hTERT and the repair reporter cells derived from HCA2-hTERT), HEK293T and U-2 OS cells were cultured in DMEM medium supplemented with 10% FBS (Gibco), 1% non-essential amino acids (Gibco), and 1% penicillin/streptomycin (Gibco), at 37˚C in a cell incubator with 5% CO_2. Human fibroblasts were transfected on a Lonza 4D nucleofector system with DT-130 program, U-2 OS cells were transfected on a Lonza 4D nucleofector system with CM-104 program, and HEK293T cells were transfected with the PEI reagent.

Mice

C57BL/6 mice were housed in a specific pathogen-free (SPF)-grade environment and exposed to a 12-hour light/dark cycle. All animal experiments were conducted in accordance with the Health Guide for the Care and Use of Laboratory Animals, and were approved by the Biological Research Ethics Committee of Tongji University.

Cell treatment

For immunostaining or repair efficiency analysis, cells were pretreated with Nu7026 (7 μM) or SCR7 (50 μM) for 12 hours to inhibit NHEJ. For CHK2 inhibition, cells were pretreated with BML-277 (10 μM) for 12 hours. Nu7026,

SCR7 and BML-277 were re-supplemented into the culture medium after cells were transfected or irradiated. Cells were irradiated with a Rad Source RS2000 system and then subjected to immunofluorescent staining, co-immunoprecipitation assay or whole cell lysate extraction. For ATM inhibition, cells were treated with KU-55933 (10 μM) for 12 hours before being lysed for co-IP. For laser micro-irradiation assay, cells were micro-irradiated with a Leica DM6500 confocal microscopy 405 nm laser diode system.

Co-immunoprecipitation

HEK293T cells transfected with indicated plasmids were harvested and lysed with RIPA buffer for 30 min on ice. The cell lysate was then sonicated at 10% duty for 3 min, and centrifuged at 13,500 r/min for 15 min at 4°C. The supernatant was then collected, and a fraction of sample was used as Input, while the rest was subjected to immunoprecipitation with the indicated antibody-conjugated beads at 4°C overnight. For nucleic acids digestion, the lysate was incubated with DNase I and RNase A for 1 hour at 37°C before proceeding with immunoprecipitation. The beads were rinsed with RIPA buffer 4 times and boiled for 10 min with sample buffer, prior to Western blot analysis.

Antibodies

The following antibodies were used in this study: anti-HA (Cell signaling, Cat. # 2367), anti-Flag (ABclonal, Cat. #AE005), anti-GFP (ABclonal, Cat. #AE012), anti-BMAL1 (ABclonal, Cat. #A4714), anti-CLOCK (ABclonal, Cat. #A7265), anti-53BP1 (Cell signaling, Cat. #4937), anti-XRCC4 (ABclonal, Cat. #A1677), anti-Lig4 (ABclonal, Cat. # A1743), anti-γH2AX (Cell signaling, Cat. #9718), anti-p16 (Abcam, Cat. #ab108349), anti-p21 (Abcam, Cat. #ab109199), anti-GAPDH (Proteintech, Cat. #60004) and anti-TUBULIN (Bioworld, Cat. #AP0064).

Repair efficiency analysis

Double-strand break repair efficiency was analyzed by using the HCA2-D4A cells integrated with the dual-fluorescence HR-NHEJ reporter and the NHEJ-I9A cells integrated with the GFP-based reporter, as previously reported. For HCA2-D4A cells, 1×10^6 cells were transfected with 5 μg of vectors encoding the I-SceI endonuclease together with 15 ng of vectors encoding mTagBFP2. For NHEJ-I9A cells, 1×10^6 cells were transfected with 5 μg of vectors encoding I-SceI and 15 ng of vectors encoding DsRed. For the CHK2 inhibition experiment, cells were pretreated with CHK2 inhibitor, BML-277, at 24-hour prior to transfection, and BML-277 was re-supplemented into the culture medium post transfection. Cells were collected for repair efficiency analysis on a FACS Verse system (BD Biosciences) 48-hour post transfection, and the raw FACS data were analyzed with FlowJo software.

NHEJ fidelity analysis

NHEJ fidelity was assessed using NHEJ-I9A cells. Cells were transfected with 5 μg of vectors encoding I-SceI and 5 μg of vectors encoding either CLOCK or BMAL1, or an empty vector. GFP positive cells were sorted using BD FACS Aria II at 72 h post transfection. Genomic DNA was subsequently extracted from the sorted cells, followed by PCR amplification using primers F: 5'-TAGACCAC TGGATTCAGAAGCGATC-3'; R: 5'-CATGAGAACCCACAGTGTTTCAGGT-3'. The resulting PCR products were purified and ligated into the pClone007 Blunt v ector (Tsingke, Cat. #TSV-007VS) for Sanger sequencing.

Comet assay

HCA2-hTERT cells were transfected with vectors encoding BMAL1 and CLOCK 48 hours prior to alkaline comet assay analysis. The detailed procedures of comet assay were according to the instructions of the

manufacturer of a comet assay kit (Trevigen, Cat. #4250-050-K). CaspLab software was employed for tail moment analysis. At least 50 cells were included for each group.

Real-time qPCR

The real-time qPCR was performed on a ViiA 7 Real-Time PCR system (Applied Biosystems) with SYBR Green reagent (Roche). The primers used for qPCR were as followed: *CDKN1A* F: TGTCCGTCAGAACCCATGC; R: AAAGT CGAAGTTCCATCGCTC; *IL6* F: GCCCAGCTATGAACTCCTTCT; R: GAAGGC AGCAGGCAACAC; *IL1b* F: CTGTCCTGCGTGTTGAAAGA; R: TTGGGTAATT TTTGGGATCTACA; *IL8* F: AGACAGCAGAGCACACAAGC; R: ATGGTTCCTT CCGGTGGT; *CCL2* F: AGTCTCTGCCGCCCTTCT; R: GTGACTGGGGCATT GATTG; *CXCL2* F: CCCATGGTTAAGAAAATCATCG; R: CTTCAGGAACAGC CACCAAT; *MMP1* F: TTTGATGGACCTGGAGGAAATC; R: TGAGCATCCCCT CCAATACC; *PER1* F: AGTCCGTCTTCTGCCGTATCA; R: AGCTTCGTAACC CGAATGGAT; *PER2* F: CTTCAGCGATGCCAAGTTTGT; R: CGGATTTCATTCTC GTGGCTTT; *NR1D2* F: TTTAGTGGCATGGTTCTACTGTG; R: AGCCTTCGCA AGCATGAACT; *TUBA1A* F: CTTCGTCTCCGCCATCAG; R: TTGCCAATCTGG ACACCA.

Senescence-associated β-galactosidase staining

Cells transfected with empty vector, or vectors encoding BMAL1 or CLOCK, were irradiated with 10 Gy X-irradiation to induce stress-induced premature senescence. β-galactosidase staining was performed at 10 days post-irradiation as previously reported. In brief, cells were fixed with a mixture of 2% formaldehyde and 0.2% glutaraldehyde for a duration of 5 minutes at room temperature. Following fixation, cells were rinsed twice with PBS. Subsequently, staining solution containing 1 mg/mL X-gal was added to the cells and incubated at 37°C overnight.

Statistical analysis

Prism GraphPad software was used for statistical analysis. The methods used for statistical analysis are indicated in the figure legends. The precise *P* value is shown in the figure.

Supplementary Figure Legends

Supplementary Figure 1. CLOCK and BMAL1 promote DSB repair.

(A) Schematic illustration of the GFP-based NHEJ reporter. (B) The effect of CLOCK and BMAL1 overexpression on NHEJ efficiency, assayed with the NHEJ-I9A system. (C) The effect of CLOCK and BMAL1 overexpression on NHEJ fidelity. (D) The effect of CLOCK overexpression on HR efficiency, assayed with the dual-fluorescence HR-NHEJ reporter. (E) The effect of BMAL1 overexpression on HR efficiency, assayed with the dual-fluorescence HR-NHEJ reporter. (F, G) Representative images showing BMAL1 and CLOCK localized at DNA damage site generated by laser micro-irradiation in U-2 OS cells (scale bar: 10 μm). Student's *t* test was performed to determine the significance of the results.

Supplementary Figure 2. CLOCK interacts with CHK2.

(A) Co-IP analysis of CLOCK–CHK2 interaction with or without DNase I and RNase A treatment. (B) The effect of ATM inhibitor (KU-55933) treatment on CLOCK–CHK2 interaction in HEK293T cells. Cells were treated with 10 μM KU-55933 before being lysed for co-IP analysis.

Supplementary Figure 3. CHK2 inhibition does not influence the canonical function of CLOCK and BMAL1 as transcriptional factors.

(A) The effect of BML-277 treatment on the mRNA level of *PER1* in HEK293T cells. (B) The effect of BML-277 treatment on the mRNA level of *PER2* in HEK293T cells. (C) The effect of BML-277 treatment on the mRNA level of *NR1D2* in HEK293T cells. The mRNA level of *TUBA1A* gene was used as the internal control. Student's *t* test was performed to determine the significance of the results.

Supplementary Figure 4. CLOCK and BMAL1 overexpression have no obvious impact on 53BP1 kinetics.

Representative images of the 53BP1 fluorescence immunostaining in HCA2-hTERT cells. Cells were treated with 2 Gy X-irradiation, and harvested for immunostaining with DAPI (blue) and anti-53BP1 (green) at the indicated timepoint (scale bar: 10 μm).

Supplementary Figure 5. Immunoblotting analysis of the expression of CLOCK and BMAL1.

Western blotting image showing the expression level of CLOCK and BMAL1. Cells were transfected with 8 μg of EV, or 4 μg of vectors encoding CLOCK together with 4 μg of EV, or 4 μg of vectors encoding BMAL1 together with 4 μg of EV, or 4 μg of vectors encoding CLOCK together with 4 μg of vectors encoding BMAL1, respectively.

Supplementary Figure 6. CLOCK overexpression reduces the mRNA levels of SASP factors.

(A) The effect of CLOCK and BMAL1 overexpression on the mRNA level of IL1b in SIPS HCA2-hTERT cells. (B) The effect of CLOCK and BMAL1 overexpression on the mRNA level of IL8 in SIPS HCA2-hTERT cells. (C) The effect of CLOCK and BMAL1 overexpression on the mRNA level of MMP1 in SIPS HCA2-hTERT cells. Student's *t* test was performed for significance determination.

Supplementary Figure 7. Age-associated alterations of CLOCK and BMAL1 expression levels across mouse tissues.

(A) Age-associated change of the *Clock* mRNA levels in multiple mouse tissues. (B) Age-associated change of the *Bmal1* mRNA levels in multiple mouse

tissues. The data was extracted from Tabula Muris Senis database, and fitted using the linear regression model. (C) Quantification of the protein level of γH2AX and p21 in the kidney of young (3-month-old) versus old (24-month-old) mice. (D, E) Western blotting analysis of the protein level of CLOCK, BMAL1, γH2AX and p16 in the intestine of young (3-month-old) versus old (24-month-old) mice. Representative blots are shown in (D), and the quantification results are shown in (E). Each dot represents for one mice, and Mann-Whitney *U* test was employed for significance determination for (C) and (E).
