# Supplementary figures and images for: Circadian factors CLOCK and BMAL1 promote nonhomologous end joining and antagonize cellular senescence
Source: Life Med. 2024 Feb 4;3(2):lnae006. doi: 10.1093/lifemedi/lnae006 (PMC11749561; doi:10.1093/lifemedi/lnae006)

Figure S1

**A**

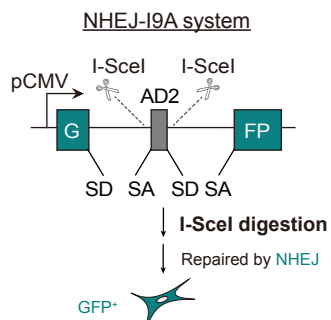

**B**

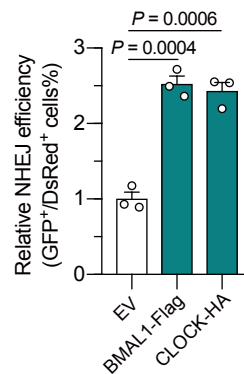

**C**

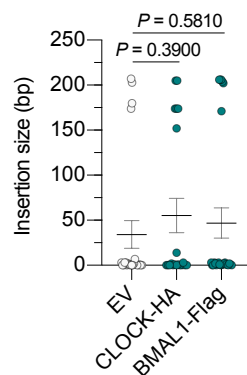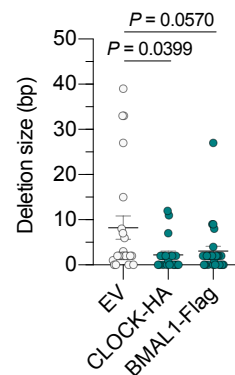

D

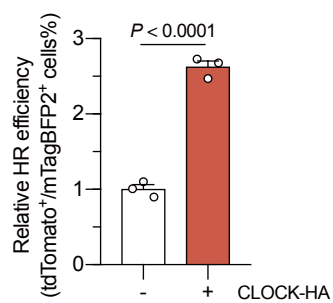

## E

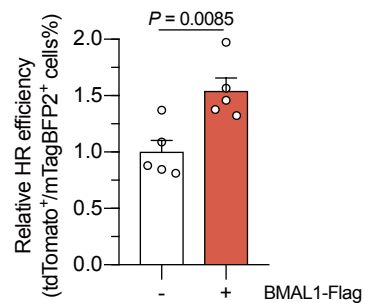

**F**

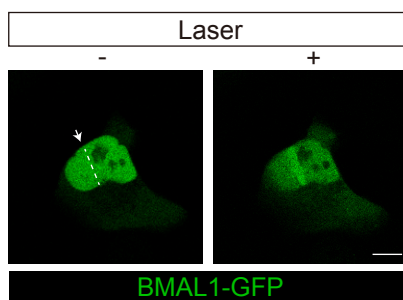

## G

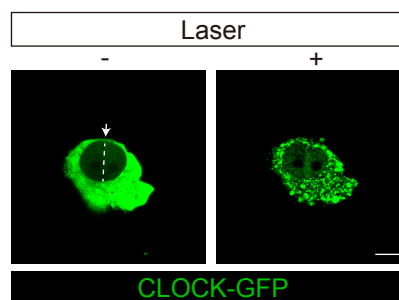

Figure S2

A

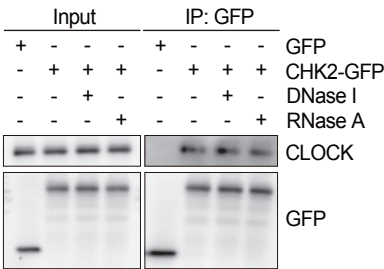

B

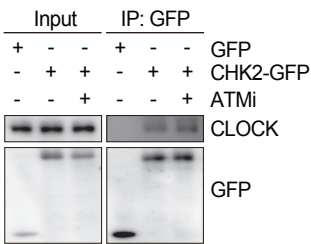

Figure S3

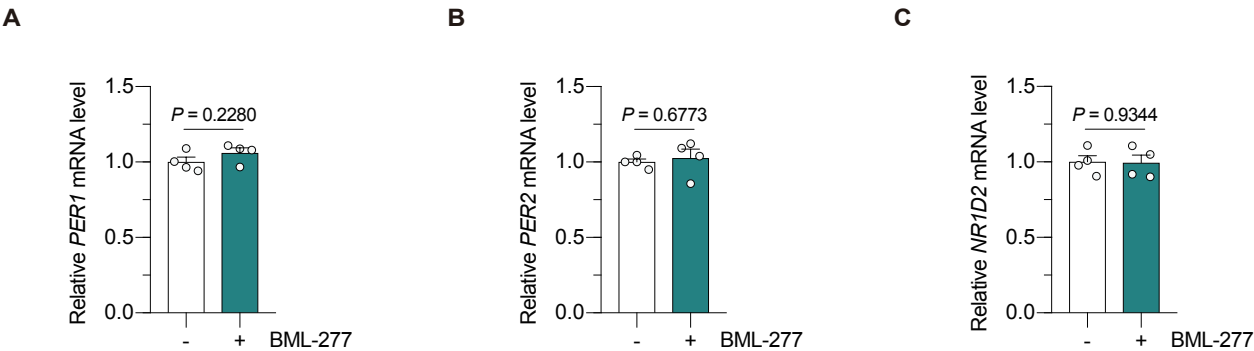

Figure S4

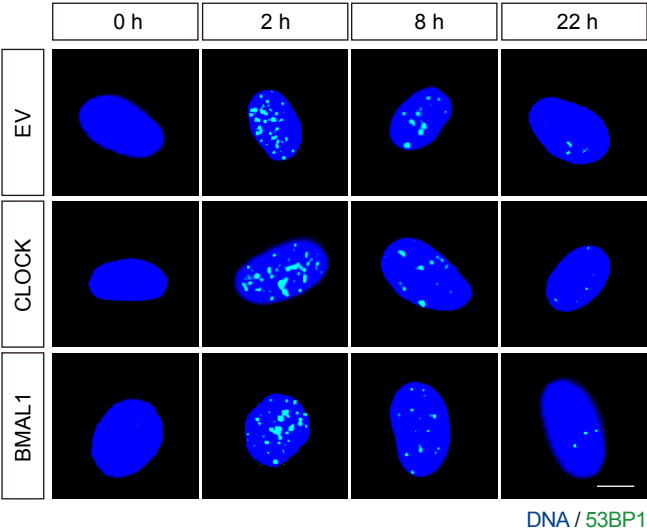

Figure S5

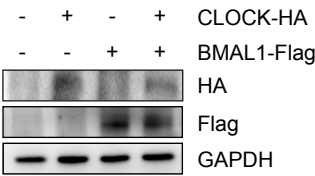

Figure S6

A

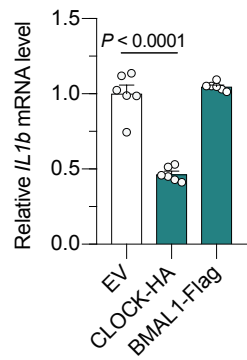

B

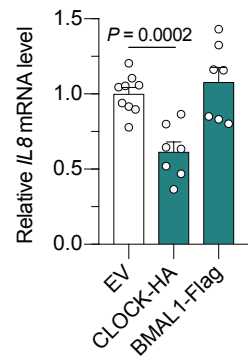

C

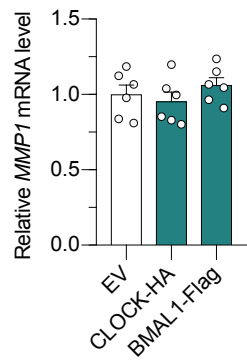

Figure S7

A

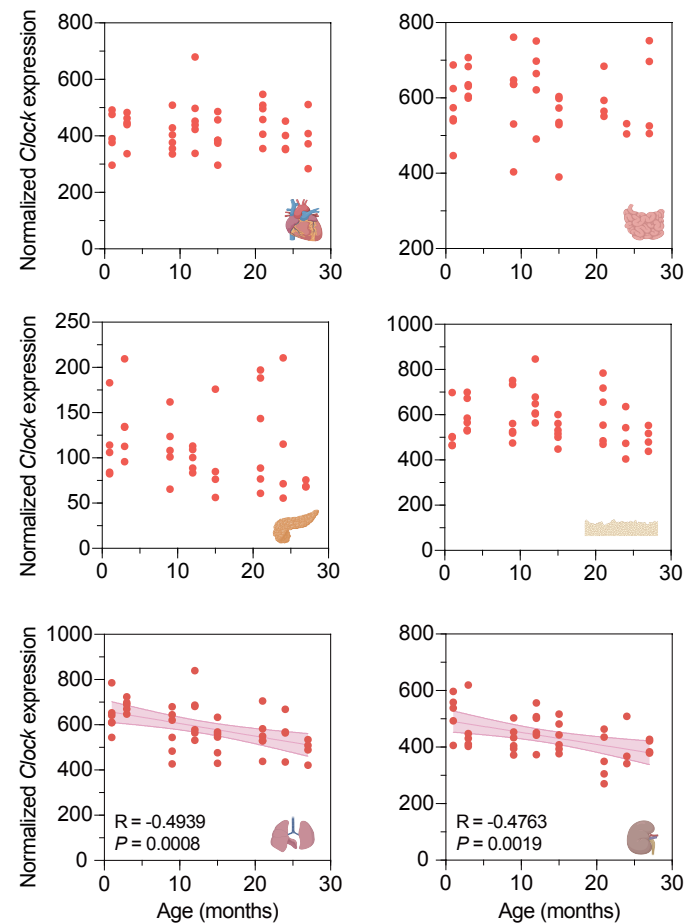

B

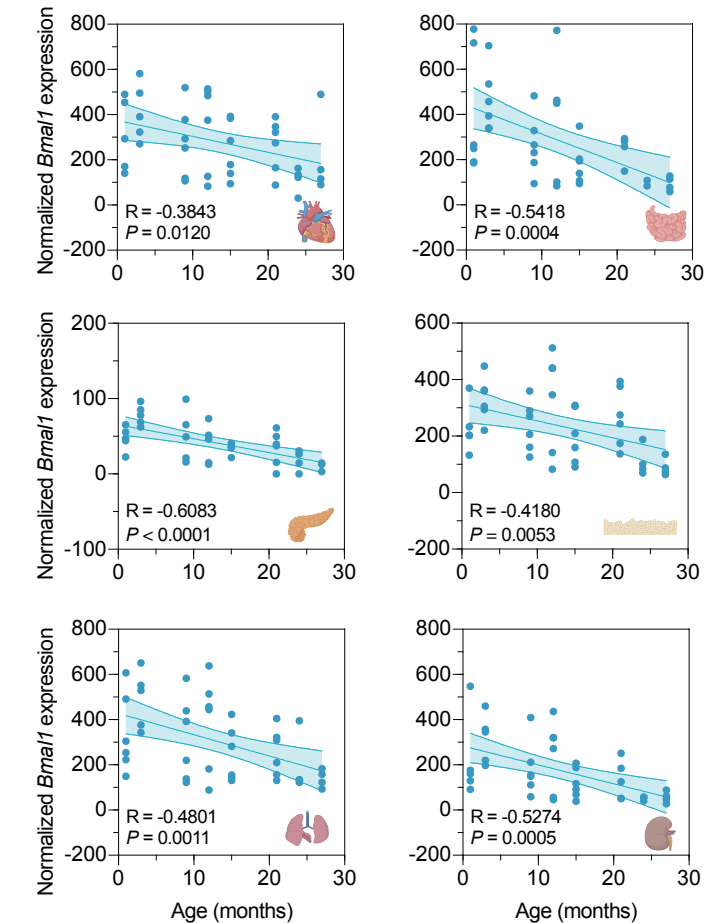

C

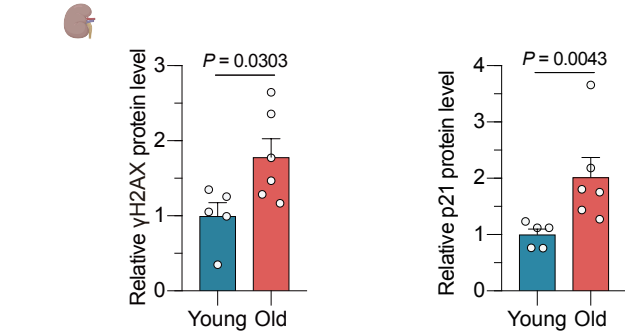

E

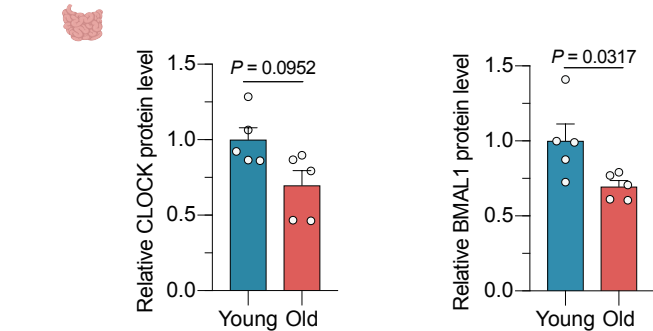

D

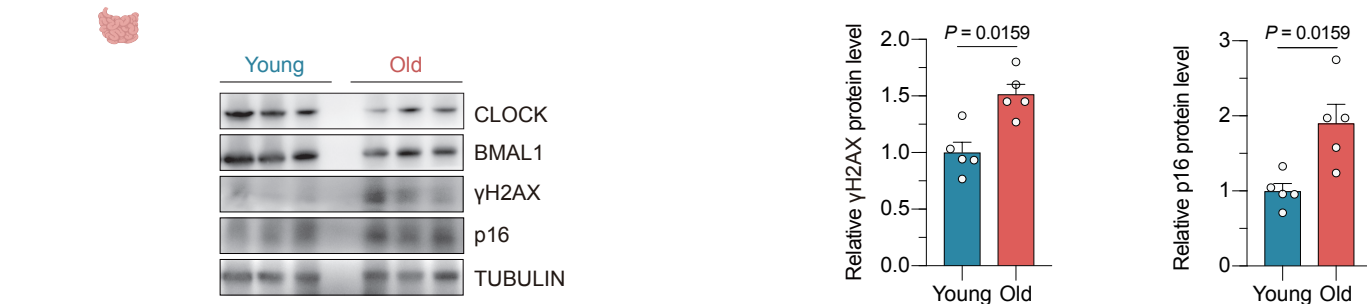

Supplement: lnae006_suppl_Supplementary_Figures_S1-S7 [file lnae006_suppl_Supplementary_Figures_S1-S7.zip › Supplementary_Figure and infomation_ready for typesetting_clean/Supplementary Figure_ready for typesetting_clean.pdf]
